# Earlier quantification of rice blast impact via instantaneous chlorophyll fluorescence

**DOI:** 10.1186/s13007-025-01391-8

**Published:** 2025-06-05

**Authors:** Insu Yeon, Jihyeon Yeo, Yejin Park, Ghiseok Kim, Jae Hoon Lee, Hyungsuk Kimm

**Affiliations:** 1 Department of Agriculture, Forestry and Bioresources, College of Agriculture and Life Sciences, Seoul National University, Seoul, 08826 South Korea; 2 Department of Agricultural Biotechnology, College of Agriculture and Life Sciences, Seoul National University, Seoul, 08826 South Korea; 3 Department of Biosystems Engineering, College of Agriculture and Life Sciences, Seoul National University, Seoul, 08826 South Korea; 4 Research Institute of Agriculture and Life Sciences, Seoul National University, Seoul, 08826 South Korea

**Keywords:** Instantaneous chlorophyll fluorescence, Rice blast, Early detection, Inoculation, Hyperspectral remote sensing, Vegetation index

## Abstract

**Background:**

Rice blast, one of the major diseases causing significant rice yield loss, downregulates the photosynthetic activity and induces aggressive spread of cell death causing food security concerns. Hence, earlier quantification of rice blast is imperative for improved management of the disease. Instantaneous chlorophyll fluorescence (e.g., sun-induced chlorophyll fluorescence under sunlight), which is mechanistically linked with photosynthesis at the photosystem scale, has shown the potential for quantifying the impact of abiotic stresses on plant physiology but remains yet to be tested for biotic stresses. Here, we assessed the potential of chlorophyll fluorescence (CF) for quantifying rice blast impact on plant physiology. In particular, we further retrieved the quantum yield of chlorophyll fluorescence (Φ_F_) by normalizing the influence of the magnitude of incident radiation.

**Results:**

Φ_F_ sensitively responded to rice blast within 24 and 96 hours post-inoculation for susceptible and resistant cultivars, respectively. We confirmed that the Φ_F_ showed strong sensitivity in response to different doses of inoculation and to cultivar difference. In addition to Φ_F_ results, we further investigated the role of red to far-red CF ratio (CF_R:FR_) in rice blast detection. CF_R:FR_, which was previously reported to be tightly coupled with chlorophyll contents, captured the impact of rice blast inoculation to some extent while green chlorophyll vegetation index did not show any difference across all inoculated groups.

**Conclusions:**

We confirmed that the Φ_F_ sensitively responded to rice blast inoculation and differentiated two dose levels of inoculation and low- and high-resistance levels via the comparison of two cultivars. Furthermore, the full spectrum of chlorophyll fluorescence was used to obtain the red to far-red CF ratio and showed its capability for indicating the physiological impact of rice blast. Our findings highlight the unique role of chlorophyll fluorescence in sensitively quantifying rice blast impact. Our approach is highly scalable through sun-induced chlorophyll fluorescence observations and thus will contribute to improving the large-scale management of rice blast.

**Supplementary Information:**

The online version contains supplementary material available at 10.1186/s13007-025-01391-8.

## Introduction

Rice blast is the most destructive disease of rice, causing significant yield losses ranging from 10 to 35% in 85 countries [14], which can be enough to feed 60 million people [10, 37]. Rice blast is highly contagious and difficult to contain if not properly controlled at an early stage. In particular, Severns et al. [41], who studied wheat stripe rust, highlighted the importance of early-stage treatment of plant diseases reporting >80% eradication when treated early enough (<1.125 latent period). Reflectance-based vegetation indices (VIs) have been studied for rice blast detection based upon the relevance of spectral reflectance to the structural and physiological status of plant leaves [9, 34]. Although VIs were effective in scoring rice blast development and quantifying early changes in leaf chemical composition, VIs did not change until chlorophyll degradation due to the lack of changes of spectral reflectance in the critical wavebands such as near-infrared (NIR), shortwave infrared, and red [33, 47]. Machine learning algorithms might improve the hyperspectral reflectance-based plant disease detection [5, 13, 22] but are still ineffective before chemical compositional changes.

Instantaneous chlorophyll fluorescence has emerged as a proxy for asymptomatic plant stress (i.e., without changes in physical and chemical structure). Chlorophyll fluorescence (CF) is a plant-emitted light signal, and when induced by solar radiation, it is well known as sun-induced chlorophyll fluorescence (SIF), yet its capability for stress detection is largely limited to abiotic stresses [26, 27, 51, 52, 56]. Early stress detection using SIF is due to its mechanistic link to the light-harvesting process and subsequent photosynthetic electron transport through the photosystems [38]. Based on such link, studies found that SIF can effectively quantify photosynthetic depressions by abiotic stresses when non-physiological variability was eliminated [26, 27, 58]. Despite emerging interest in the potential of CF, it remains uncertain whether CF can also effectively quantify biotic stress, particularly, a disease impact. Since rice blast depresses the photosynthetic activity, CF could retain its ability to capture the effect of rice blast in the asymptomatic period [1, 15].

For plant physiological investigations, one of the spotlighted aspects of CF is in the ratio of CF at red bands to CF at far-red bands (CF_R:FR_). The usefulness of CF_R:FR_ at leaf level results from its link to leaf chlorophyll contents as reabsorption in CF_Red_ increases with the increase of chlorophyll content [2, 19]. Recent studies also suggested that the C_FR:FR_ is an effective indicator for water and salt stress at leaf scale and for nitrogen at both leaf and canopy scales [43, 55]. Moreover, under drought stress, CF_R:FR_ was found to be largely regulated by the maximum potential electron transport rate, not solely by chlorophyll contents [62]. Thus if rice blast affects leaf structure or subtle spectral absorption and maximum potential electron transport rate for CF during the asymptomatic stage, the full spectrum of CF bands would further contribute to the early detection of rice blast.

In this study, we conducted an indoor experiment of rice blast inoculation and aimed to assess early detection of physiological depression in asymptomatic stage through hyperspectral spectroscopy and full spectrum CF. Specifically, here we focus on the following science questions: (1) How does CF compare to spectral reflectance-based VIs in quantifying the impact of rice blast during its asymptomatic stage? (2) How sensitively does CF respond to rice blast inoculation across inoculation intensity levels and cultivars with different resistance levels? (3) What added value does the full spectrum of CF provide in quantifying rice blast impact? For answering the questions, we inoculated rice blast at low and high concentrations for a susceptible and resistant cultivar in an environment-controlled greenhouse and collected hyperspectral reflectance spectra and CF spectra using a hyperspectral spectroscopic system. Our result will be the first demonstration of quantifying rice blast impact in its asymptomatic stage using CF. Earlier detection of rice blast will allow for more effective control of epidemics substantially contributing to reliable crop production and food security.

## Materials and methods

### Rice blast inoculation

We raised rice seedlings for 28 days since germination (or until 4–5-leaf stage) in a growth chamber under controlled conditions (air temperature: 27–28 °C; relative humidity (RH): 50–60%; photosynthetic photon flux density: 180 μmol m^−2^ s^−1^). To address how inoculation intensity and cultivar resistance influence the CF response to rice blast infection, we designed a factorial experiment using two rice cultivars with contrasting resistance levels—Hopyeong (susceptible) and Dasan (resistant). The experiment consisted of total 18 pots, with 9 pots for each cultivar. For each cultivar, 3 pots were mock inoculation group, another 3 pots were low-concentration group, and the rest 3 pots were high-concentration group.

Rice blast fungus (*Magnaporthe oryzae*, KJ201) were subcultured for 7 days at agar plates under a lighted condition. Cultured fungus were collected and used to prepare spore suspension for spray inoculation. We conducted the spray inoculation on to the subject rice at a low (5⋅10^3^ conidia/mL) and high concentration (5⋅10^5^ conidia/mL) and immediately bagged the entire shoots in a plastic bag for maintaining very humid condition (RH nearly 100%). For successful inoculation, the rice plants were kept in a growth chamber with the plastic bag for 24 h under a dark condition. Rice blast inoculation was performed on February 17, 2023. To promote rice blast infection and colonization, greenhouse RH was increased to >70% since the time of inoculation, and seedlings were kept under a stable humid environmental condition for 48 h after the inoculation.

### Assessment of rice blast progress

**Fig. 1:**
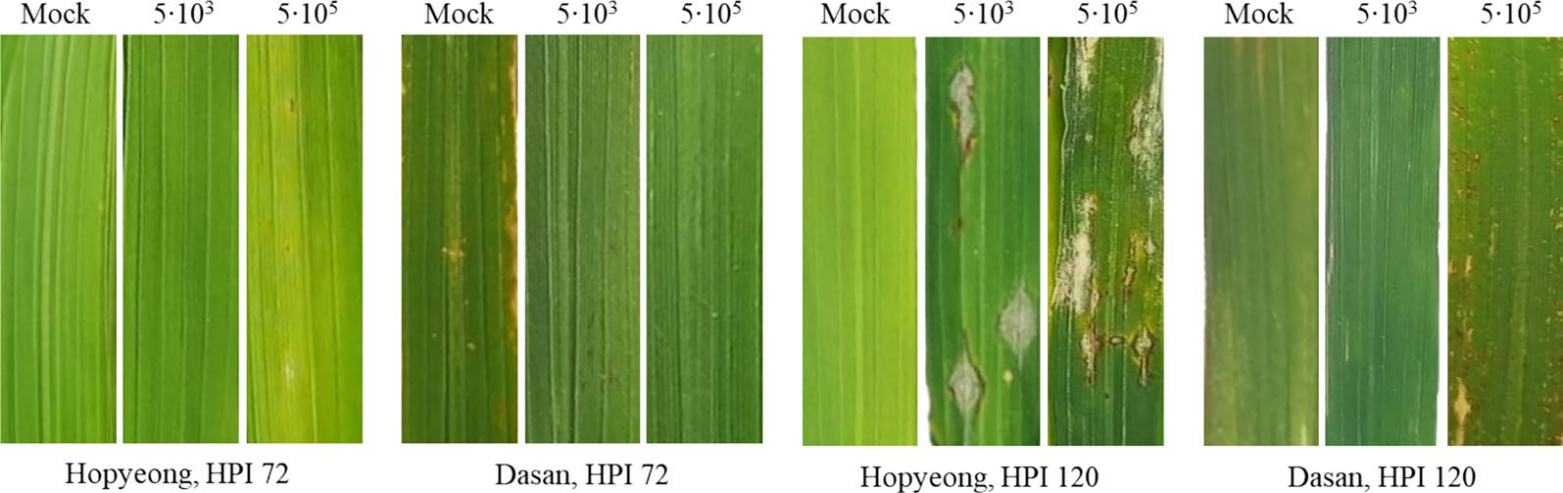
RGB images of rice leaves of Hopyeong (rice blast-susceptible cultivar) and Dasan (rice blast-resistant cultivar) in 72 and 120 hours post-inoculation (hpi). Each image was labeled with inoculation severities. We note that mock group leaves of Dasan cultivar exhibited vague brown or yellow stains, which are due to drying and irrelevant to rice blast

We assessed rice blast infection progress following rice leaf blast scoring criterion on a 0–9 rating scale given by International Rice Research Institute [23]. On this scale, 0 represents no visible lesions, 1 indicates the presence of small brown specks of lesion of pinpoint size, 2 refers to leaves showing small roundish necrotic gray spots about 1–2 mm in diameter, 4 represents leaves exhibiting diamond-shape lesion covering less than 4% area, and 9 indicates that more than 75% leaf area is affected (Table S1). We randomly selected 5–8 leaves within each inoculated group to score disease progression. For the reliable evaluation, the assessment of infection progress was conducted based on RGB images (Fig. 1). To minimize the potential impacts that may occur on the leaves during photography, we collect RGB images in the greenhouse immediately after the hyperspectral measurements.

### Hyperspectral spectroscopic measurements

We collected hyperspectral spectroscopic data from the leaves of rice seedling. We conducted leaf scale measurements for 9 days in total with 4 consecutive days for pre-inoculation measurements (−96, −72, −48, −24 hours post-inoculation; hpi) and 5 consecutive days for post-inoculation measurements (24, 48, 72, 96, 120 hpi). To exclude the impact of diurnal variation of CF, we conducted measurements at the fixed time window 10:30 AM to 12:00 PM, where we could retrieve maximal CF [6, 59]. We randomly selected 4–8 leaves from each group for spectral reflectance and CF spectrum measurements on each day. We confirmed the initial stage of the disease spread at 72 hpi and further expansion through 120 hpi, and we only took measurements from green areas (non-lesion areas) to assess the unique role of CF for quantifying the rice blast impact while maintaining green where leaf structure remains unchanged.

For the measurements, rice seedlings were moved from the growth chamber to an indoor greenhouse (73*142*195; W*D*H cm) built in a laboratory. To avoid environmental stresses, the greenhouse environment was maintained at a temperature range of 27–28 °C and a relative humidity >70%. We used a spectroscopic system with two spectrometers, QEpro and HR2000+ (Ocean Optics, Dunedin, FL., USA) with the same configuration and specification as the system used in Yeo et al. [54]. To conduct leaf-scale measurements with the system, we used two CLP-040001 leaf clips (Spectral Evolution, Haverhill, MA., USA) for the two spectrometers. The leaf clips contained a 5 W tungsten halogen lamp and two reference plates, a white reference plate (~100% reflectance) on one side and black reference plate on the other side. For the leaf clip connected to the QEpro by a fiber optic cable, we attached an FLD 601 DSP bandpass filter (Iridian Spectral Technologies, Ottawa, ON., CA) to the light inlet inside the leaf clip for measuring the full spectrum of CF. The filter almost completely blocks light in the 608–1000 nm (<3% transmittance) and allows for efficient light transmittance (transmittance > 90%) at shorter wavelength (<594 nm). As the bandpass filter blocked the incident light at the wavelength range for the CF spectrum (650–850 nm), the QEpro directly quantified the magnitude of the full spectrum of CF (Fig. S1).

### Hyperspectral data analysis

To quantify the structural impact of rice blast on leaf tissues, we calculated tow reflectance-based vegetation indices: the Green chlorophyll vegetation index (GCVI), a proxy for leaf chlorophyll content [20], and Photochemical reflectance index (PRI), which is significantly correlated with carotenoid/chlorophyll ratio at leaf level [17, 44]. GCVI and PRI were calculated as follow:

1 $$GCVI = \frac{\rho NIR}{{\rho Green}} - 1$$

2 $$PRI = \frac{\rho 531 - \rho 570}{{\rho 531 + \rho 570}}$$

where $\rho Green$ and $\rho NIR$ denote the green (540–560 nm) and NIR (770–780 nm) reflectance, respectively. $\rho 531$ and $\rho 570$ are the reflectance at 531 nm and 570 nm, respectively.

To capture the physiological downregulation caused by rice blast, we obtained full spectrum of CF by applying the bandpass filter to a leaf clip connected to QEpro. The filter still transmitted ~3% of incoming NIR radiation, which contributed to the obtained CF spectrum after the reflection on leaf surface. To account for the contribution of the filter-transmitted NIR radiation in the upwelling radiance, we quantified the incoming radiance transmitted through bandpass filter at Red and NIR bands (660–780 nm) and $\rho NIR$, which is measured by HR2000+. Here we presumed that the NIR radiation of the leaf clip’s light source is large enough to ignore the contribution of CF in the upwelling radiance as well as $\rho NIR$ measured by HR2000+. The leaf surface-reflected NIR radiation was estimated as the product of the transmitted radiance and the reflectance, and then we subtracted that from the upwelling radiance to obtain pure CF spectrum.

3 $$CF\left( \lambda \right) = L_{F,Up} \left( \lambda \right) - L_{F,Down} \left( \lambda \right)*\rho NIR\left( \lambda \right)$$

We note that *L*_*F,Up*_ and *L*_*F,Down*_ represent upwelling radiance from the leaf and downwelling radiance from the light source, respectively. The upwelling radiance includes both reflected radiance from the leaf and the chlorophyll fluorescence emitted by the leaf, whereas the downwelling radiance corresponds to the direct incident radiation provided by the light source. We then applied a framework suggested by Dechant et al. [12], Zeng et al. [57], and Guanter et al. [21] to retrieve the physiological signal and to remove non-physiological signals (i.e. radiation and canopy structure) from the CF as follow:

4 $$CF\left( \lambda \right) = PAR \times fAPAR_{chl} \times f_{esc} \times \Phi_{F} \left( \lambda \right)$$

The CF can be disaggregated into radiation (Photosynthetically active radiation, PAR), the fraction of chlorophyll-absorbed PAR (fAPAR_chl_), the escape ratio from the canopy (f_esc_) and physiological CF yield (Φ_F_). The Φ_F_, closely related to plant physiological mechanisms, does not contain radiative or canopy structural information, thus can be a potential physiological stress indicator in our study. In the derivation of Φ_F_, we excluded f_esc_ which solely represents canopy structural information [12], as the measurements were conducted at the leaf level. In principle, fAPAR_chl_ is influenced by both chlorophyll contents and canopy structure. However, the impact of canopy structure on fAPAR_chl_ was found to be as significant while that of chlorophyll contents was relatively small [29], therefore, we ignored fAPAR_chl_ in the calculation for Φ_F_. In Eq. (4), PAR can be replaced with red band radiance (Red) as it is stably proportional to the incoming PAR. With these rationales, we can simplify the equation for Φ_F_ as follow:

5 $$\Phi_{F} \left( \lambda \right) \approx \frac{{{\text{CF}}\left( \lambda \right)}}{{L_{Red} }}$$

where L_Red_ denotes the mean of the red radiance (644–648 nm). In this study, we will henceforth refer to $\frac{{{\text{CF}}}}{{L_{Red} }}$ as Φ_F_ from now on. We, then, calculated red to far-red fluorescence ratio (CF_R:FR_) to evaluate what the full spectrum of CF can provide in quantifying the rice blast impact, following the below equation:

6 $$CF_{R:FR} = \frac{{CF_{Red} }}{{CF_{Far - Red} }}$$

CF_Red_ and CF_Far-Red_ represent mean of CF signals between 684–688 and 738–742 nm, respectively. To introduce concept of CF_R:FR_ briefly, the re-absorbance portion of CF_Red_ by chlorophyll is relatively huge compared to that of CF_Far-Red_ in canopy so that increase in chlorophyll contents leads to decrease in CF_R:FR_ [2, 53]. We provide a schematic overview of our workflow in Fig. S1.

### Statistical analysis

We evaluated the statistical significance between two variables with two-sided *t* test at a confidence level of 95%. The analysis of variance (ANOVA) was applied with post-hoc Tukey tests for multiple pair-wise comparisons to quantify the significance of differences between groups with varying intensities of inoculation. We represented significant differences in pair-wise groups using Compact Letter Display (*p* < 0.05). All statistical analyses were performed in MATLAB R2023a (MathWorks Inc., Natick, MA, USA) using core statistical functions and the Statistics and Machine Learning Toolbox for *t* tests, ANOVA, and post-hoc Tukey tests.

## Results

### Rice blast inoculation impact on spectral reflectance and CF spectrum

**Fig. 2:**
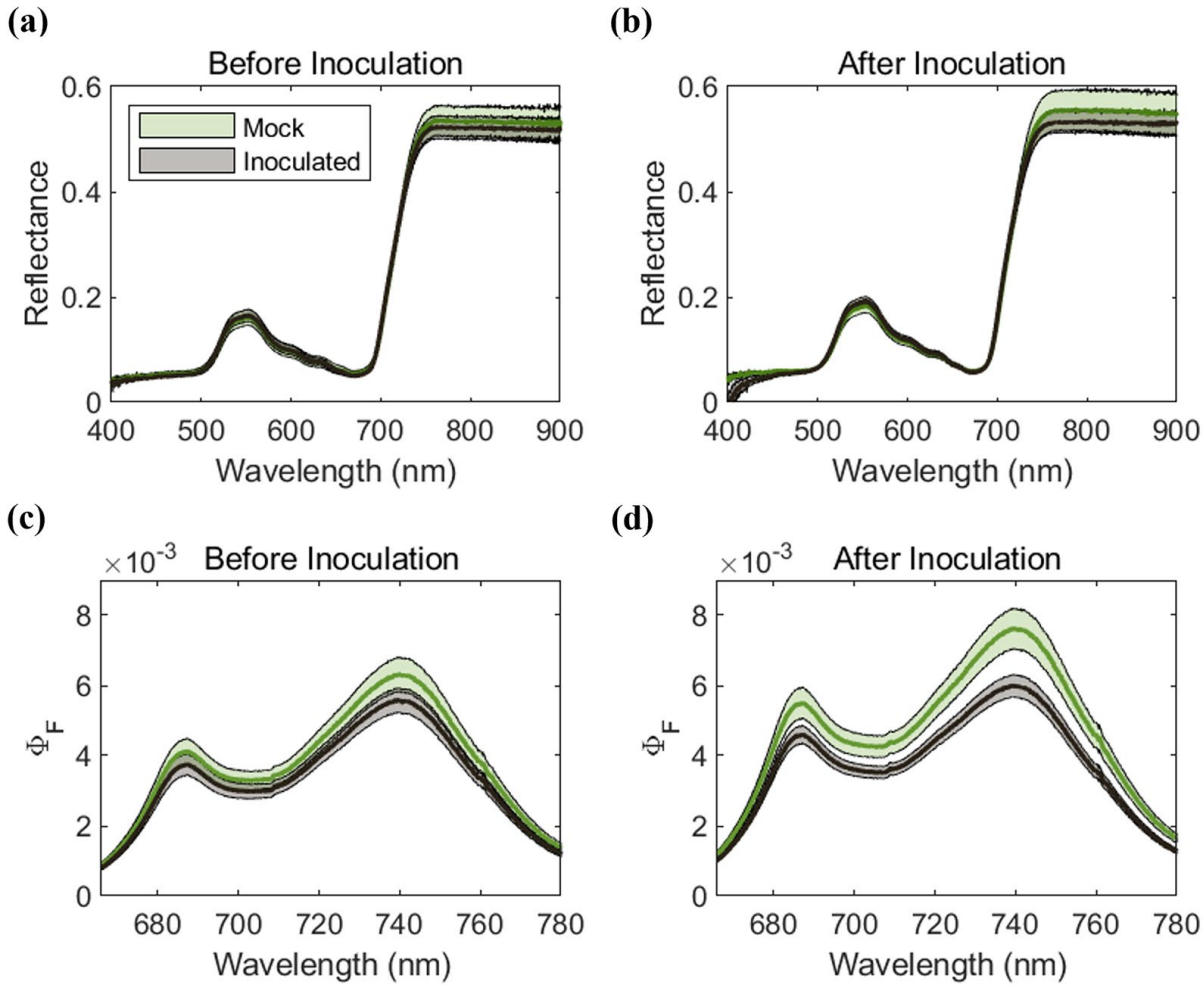
Comparisons of full spectrum of reflectance and Φ_F_ between mock and inoculated groups before (a, c) and after (b, d) inoculation. Data before inoculation was acquired from 24 to 96 h before inoculation and after inoculation from 24 to 120 hours post-inoculation (hpi). To visualize the general pattern of the inoculation impact, we combined the two cultivars for each line, and also combined the two levels of inoculation intensity for inoculated group. The green and black line indicate the average of Group_Mock_ and Group_Inoculated_, respectively. The shaded areas indicate the 95% confidence interval. The difference in Φ_F_ between mock and inoculated groups increased significantly after inoculation while reflectance showed no corresponding change (*p* < 0.05 and *p* < 0.001, respectively)

We compared the full spectrum of reflectance and Φ_F_ before and after the inoculation and only Φ_F_ spectra responded to the impact of rice blast than spectral reflectance (Fig. 2). Before the inoculation, we observed a marginal Φ_F_ difference between Group_Mock_ and Group_Inoculated_ (*p* < 0.05), and after the inoculation, the Φ_F_ difference increased and became clearer (*p* < 0.001). For spectral reflectance, we found no significant difference between the groups for most of the wavelength range (>470 nm) at both before and after the inoculation (*t* test, *p* > 0.1).

### Temporal changes after inoculation

**Fig. 3:**
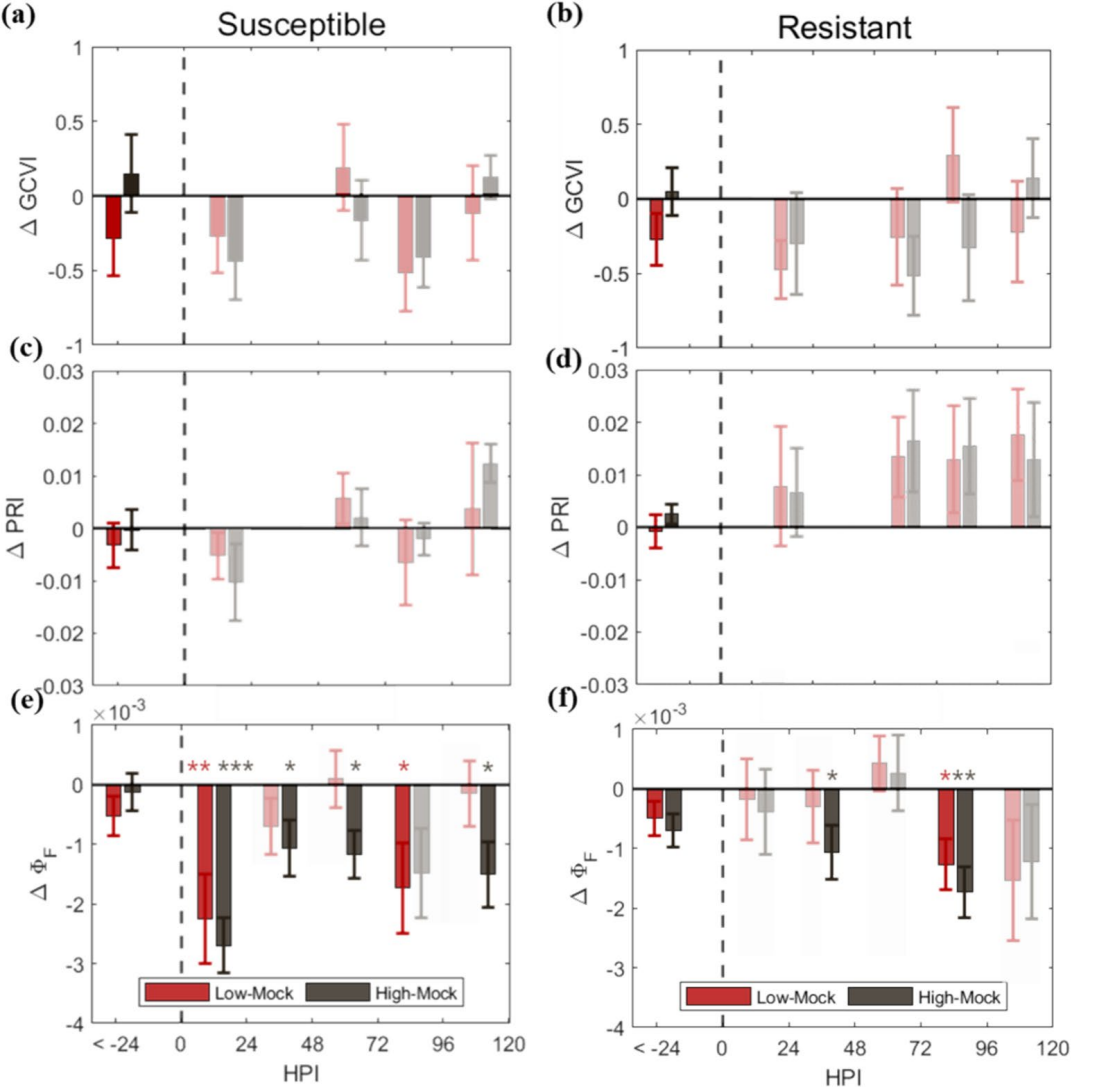
Averaged differences of Green Chlorophyll Vegetation Index, Photochemical Reflectance Index, and Φ_F_ at 760.5 nm between mock and inoculated groups at two dose levels for the susceptible cultivar (**a**, **c**, **e**) and resistant cultivar (**b**, **d**, **f**).The red bar indicates the difference of average between Group_Low_ and Group_Mock_ and the black bar represents the difference of average between Group_high_ and Group_mock_. The grey dash lines indicate the point of inoculation. The error bars indicate the standard error of differences. The asterisks shown at the top of the bars indicate significant differences between Group_Low_ and Group_Mock_ and Group_High_ and Group_Mock_ (* *p* < 0.05, ** *p* < 0.01, *** *p* < 0.001). Bars without asterisks are faded for clear visualization. Φ_F_ showed a significantly sensitive response to rice blast inoculation, particularly under high dose of inoculation in susceptible cultivars, while GCVI and PRI failed to capture any response

We analyzed temporal changes in spectral signals to assess the potential of VIs and CF for the earlier detection of rice blast (Fig. 3). We noticed that GCVI and PRI did not capture any impact of rice blast inoculation across all cultivars and doses throughout the experiment (Fig. 3a–d). On the other hand, Φ_F_ captured the impact of rice blast inoculation. For the high-dose inoculation, Φ_F_ first captured the impact at 24 and 48 hpi for the susceptible (*p* < 0.01) and resistant cultivar (*p* < 0.001), respectively (Fig. 3e, f). For the low-dose inoculation, Φ_F_ captured the impact since 24 and 96 hpi for the susceptible (*p* < 0.01) and resistant cultivar (*p* < 0.05), respectively (Fig. 3e, f). The Φ_F_ difference between mock and treatment plants appeared intermittently indicating fluctuations in the physiological response of rice as the inoculation progresses.

### Comparison between cultivars and between inoculation intensities

**Fig. 4:**
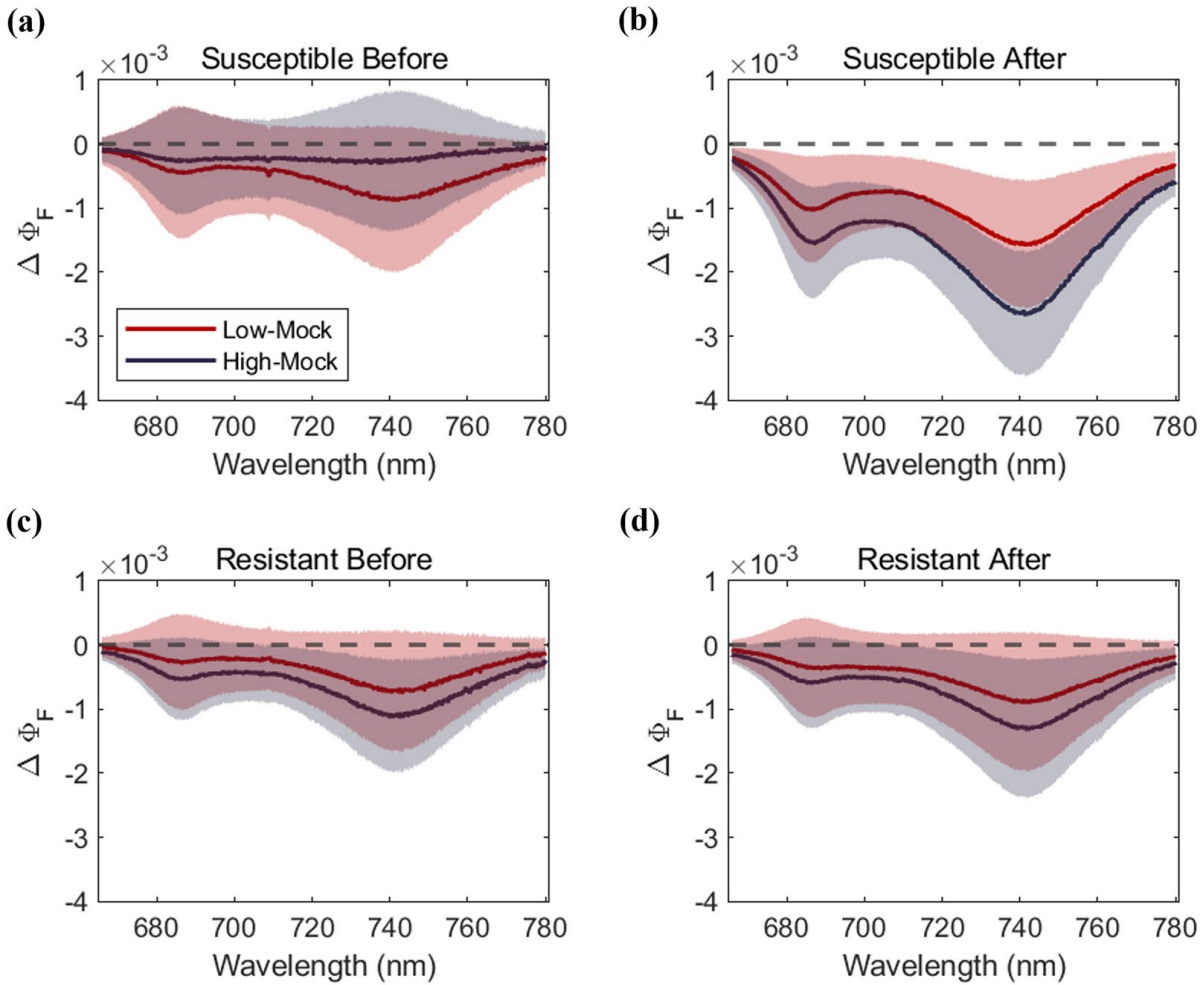
Full spectrum of Φ_F_ for the susceptible (**a**, **c**) and the resistant cultivar (**b**, **d**). Measurement data before inoculation (**a**, **b**) was acquired from −96 to −24 hpi and after inoculation (**c**, **d**) from 24 to 120 hpi. The green, red, and black lines indicate the average of Group_Mock_ with no inoculation, Group_Low_ with concentration 5⋅10^3^ conidia/mL and Group_High_ with concentration 5⋅10^5^ conidia/mL respectively, for corresponding period. The shaded areas indicate the 95% confidence interval. Φ_F_ exhibited greater sensitivity to rice blast impact in the susceptible cultivar, whereas the response in resistant cultivar remained limited

We further examined the full spectrum of Φ_F_ averaged separately for the pre-inoculation period and post-inoculation data, and assessed its potential for quantifying the impact of rice blast with different dose levels. Φ_F_ was effective in quantifying the effect of doses and the resistance levels of the cultivars. We conducted ANOVA tests on the full spectrum Φ_F_ across Group_Mock_, Group_Low_, and Group_High_ for each cultivar, analyzing pre- and post-inoculation data separately. In the susceptible cultivar, no significant difference was found before inoculation across the entire wavelength range (Fig. 4a), but after inoculation, significant differences were found at all wavelengths between the three groups (i.e., between Group_Mock_ and Group_Low_, between Group_Mock_ and Group_High_ and between Group_Low_ and Group_High_) (*p* < 0.05; Fig. 4b). For the case of the resistant cultivar, no significant differences were observed between Group_Mock_ and Group_Low_ and between Group_Low_ and Group_High_, throughout the experiment for all wavelengths (*p* > 0.05; Fig. 4c, d). Group_Mock_ and Group_High_ showed significant differences for both in pre- and post-inoculation periods (*p* < 0.05; Fig. 4c, d), indicating a potential physiological discrepancy between the groups during the pre-inoculation period. However, post-inoculation difference was clearer (difference appeared at 355 bands out of a total 375 bands within 730–780 nm, *p* < 0.05; Fig. 4d) than pre-inoculation difference (only at 34 bands, *p* < 0.05; Fig. 4c) suggesting that Φ_F_ was clearly capturing the impact of rice blast.

### Evaluation of red to far-red chlorophyll fluorescence ratio as a rice blast indicator

**Fig. 5:**
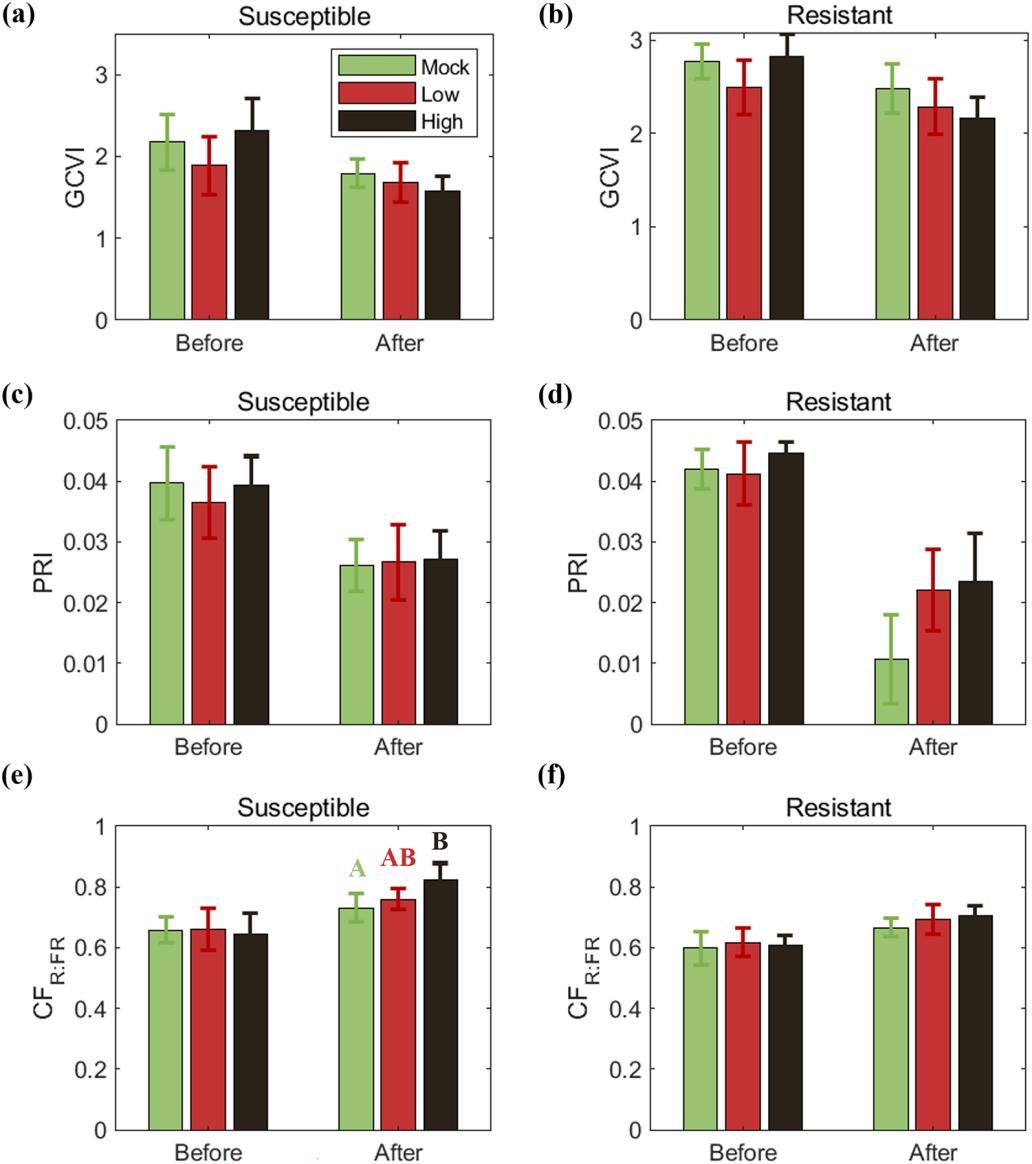
The mean of green chlorophyll index, photochemical reflectance index and red to far-red fluorescence ratio (CF_R:FR_) based on all collected data before inoculation (from −96 to −24 hpi) and after inoculation (from 24 to 120 hpi) for susceptible (**a**, **c**) and resistant cultivar (**b**, **d**). The green, red, and black boxes indicate the average of Group_Mock_ with no inoculation, Group_Low_ with concentration 5⋅10^3^ conidia/mL and Group_High_ with concentration 5⋅10^5^ conidia/mL respectively, for the corresponding period. The error bars indicate the 95% confidence interval. Compact Letter Display indicates pair-wise groups from ANOVA with post-hoc Tukey tests. CF_R:FR_ responded marginally to rice blast inoculation only in the susceptible cultivar

We evaluated the capability of CF_R:FR_ for detecting rice blast impact. Here, we tested CF_R:FR_, and found that CF_R:FR_ responded to the rice blast inoculation. For susceptible cultivar, ANOVA analysis with post-hoc Tukey tests revealed that CF_R:FR_ quantified the impact of rice blast inoculation, exhibiting an increase with higher inoculation severities after inoculation (*p* < 0.05, Fig. 5a). However, for resistant cultivar, CF_R:FR_ showed no significant change across varying inoculation concentrations (*p* > 0.05, Fig. 5b). GCVI and PRI, on the other hand, showed no significant differences (*p* > 0.05, Fig. 5c–f). The contrasting responses of CF_R:FR_ to rice blast inoculation between susceptible and resistant cultivars might be attributed to the limited sensitivity of the CF_R:FR_.

## Discussion

### How does CF compare to spectral reflectance-based VIs in quantifying the impact of rice blast during its asymptomatic stage?

VIs failed to detect any impact of rice blast while Φ_F_ detected the impact during the asymptomatic stage (within 24 and 96 hpi for susceptible and resistant cultivars, respectively; Fig. 3; Table S2). After the appearance of symptoms, Φ_F_ detected the impact of rice blast inoculation in the non-lesion area, where spectral reflectance did not show any decrease. These results suggest that Φ_F_ can detect the impact of rice blast disease earlier than VIs or spectral reflectance [9, 34, 47, 60, 61]. GCVI and PRI, that indicate chlorophyll content [18] and the ratio of chlorophyll to carotenoid [17], respectively, did not change during asymptomatic period. This suggests no detectable change occurred in chlorophyll content, which is the fastest indication of rice blast infection [47].

The observed Φ_F_ response to rice blast was faster than any other studies of using spectral reflectance-based information [40, 46, 47]. When Φ_F_ quantified the rice blast impact in this study, spectral reflectance did not exhibit any decrease. The earlier detection by Φ_F_ than VIs or other spectral signals may be explained by the high correlation between Φ_F_ and photosynthetic activity. Rice blast downregulates photosynthetic activity without altering leaf structures [1] possibly due to pathogen-secreted phytotoxins. Specifically, tenuazonic acid, which is one of the secondary metabolites produced by rice blast fungus, blocks the electron pathway of photosystem II by binding with plastoquinone B [7, 8] and supports the colonization of rice blast pathogen [11]. As lines of evidence support, Φ_F_ has the potential of quantifying such pathogen impact through the positive linear correlation between Φ_F_ and photochemical quantum yield [30, 39, 49].

### How sensitively does Φ_F_ respond to rice blast inoculation across inoculation intensity levels and cultivars with different resistance levels?

One of the notable features of Φ_F_ for rice blast detection is the strong sensitivity in response to different doses of rice blast inoculation. In the temporal analysis of Φ_F_, we found a more frequent difference in Group_High_ on 4 days for the susceptible cultivar and 2 days for the resistant cultivar, respectively. However, Φ_F_ difference in Group_Low_ only appeared on 2 days and 1 day for susceptible and resistant cultivar, respectively. Then from the full spectrum of Φ_F_ result, we observed that Φ_F_ made a distinction between Group_Mock_, Group_Low_, and Group_High_ in susceptible cultivar. Our Φ_F_ results suggest a quantitative relationship between the amount of photosynthetic downregulation and the severity of rice blast infection. Considering the importance of precise assessment of disease severity for effective disease control [24, 42], Φ_F_ can be a key to controlling rice blast.

We further explored to what extent Φ_F_ can contribute to assessing the rice blast resistance of rice cultivars. The full spectrum Φ_F_ averaged for the entire post-inoculation period revealed that Φ_F_ response to rice blast is much stronger in susceptible than resistant cultivars (Fig. 4). Without data aggregation, we found a more frequent Φ_F_ response in the susceptible cultivar than the resistant cultivar. Considering the results, we believe that Φ_F_ has the potential for assessing rice blast resistance. Studies have highlighted the importance of breeding disease-resistant crops to reduce yield losses caused by plant diseases [36, 45, 48]. Disease-resistant assessment through conventional breeding strategies (e.g. marker-assisted breeding strategies), however, is expensive and time-consuming to assess disease resistance [35], and chlorophyll fluorescence would provide faster, more efficient approaches.

### What added value does the full spectrum of CF provide in quantifying rice blast impact?

By assessing the full spectrum of Φ_F_ (Fig. 4), we confirmed that far-red Φ_F_ was more effective in quantifying the dose level difference than red Φ_F_. The different sensitivity of red Φ_F_ and far-red Φ_F_ to different dose levels may be due to spectral features of CF. The changes in the spectral shape of red CF are largely attributed to chlorophyll contents while far-red CF is less affected by chlorophyll contents. Magney et al. [32] demonstrated that the shape of red CF under water stress conditions is primarily dominated by the variation of chlorophyll content across 27 species, i.e., less sensitive to non-structural, physiological stresses. Similarly, we also found that red Φ_F_ did not sensitively respond to rice blast, likely due to the lack of GCVI sensitivity to rice blast where GCVI is a reliable proxy for leaf chlorophyll content [20].

The full spectrum CF was effective in testing the ratio index, CF_R:FR_. While previous studies reported that CF_R:FR_ is indicative of chlorophyll content and photosynthetic activity [2, 62], our results showed that CF_R:FR_ differences between the groups were irrelevant to GCVI or PRI (Fig. 5). CF_R:FR_ is often affected by re-absorption of red fluorescence in leaf structure, but it is unlikely in our result. This implies that CF_R:FR_ might contain unique information on physiological stress due to the rice blast infection. In the comparison between the cultivars and dose levels, we found that CF_R:FR_ was sensitive enough to differentiate the cultivar difference and dose level difference, but the responses were not as clear as we confirmed from Φ_F_ result. Despite the limited potential of the ratio index, our findings warrants further research to fully understand its capability and limitations.

### Implications for larger scale applications

To our best knowledge, this is the first demonstration of the potential of the Φ_F_ for rice blast detection compared to conventional reflectance-based VIs, which often failed to capture physiological stress during asymptomatic stages due to their reliance on structural or pigment-related changes. Although several studies have shown the use of CF for detecting biotic stresses through Pulse-Amplitude-Modulated (PAM) measurements [3, 4], PAM-based CF requires a strictly controlled condition where lighting modulation protocol is allowed and is inherently limited to leaf scale applications [25, 28, 31]. On the contrary, our approach simply quantifies the instantaneous intensity of CF and makes it directly compatible with currently available operational SIF measurements at different spatial scales from fields to the satellites [16, 26, 27]. With recent advances in SIF retrieval algorithms demonstrating reliable Φ_F_ retrieved under FLEX mission configurations [50], the satellite-based detection of rice blast became more feasible.

## Conclusion

We assessed the potential of CF, particularly fluorescence yield (Φ_F_), for the detection of rice blast in its asymptomatic period. Φ_F_ successfully captured the impact of rice blast within 24 hpi, even in the absence of any changes in spectral reflectance as well as vegetation indices. These results highlight that the physiological information contained in CF can allow for earlier detection of rice blast in prior to structural changes in leaf tissues. We further addressed the sensitivity of Φ_F_ in distinguishing the severities of the inoculation and resistance levels of two cultivars. Our study also investigated the utilization of full spectrum CF and suggested that the CF ratio index (CF_R:FR_) was effective in detecting rice blast impact. Though the application of CF_R:FR_ was not as effective as Φ_F_, further studies would be needed to determine the importance of the information contained in the full spectrum CF. With these findings, this study highlights the potential of implementing field- and satellite-level Φ_F_ observations for the real-time monitoring of rice blast outbreaks, although this remains to be further examined.. Furthermore, our approach will contribute to the broader applications of biotic stress detection in crops.

## Supplementary Information

**Additional file 1.**

## Data Availability

No datasets were generated or analysed during the current study.

## References

[1] Bastiaans L. Effects of leaf blast on photosynthesis of rice. 1. Leaf photosynthesis. Neth J Plant Pathol. 1993;99(4):197–203. 10.1007/BF01974664.

[2] Buschmann C. Variability and application of the chlorophyll fluorescence emission ratio red/far-red of leaves. Photosynth Res. 2007;92(2):261–71. 10.1007/S11120-007-9187-8. 17525834 10.1007/s11120-007-9187-8

[3] Calderón Madrid R, Navas Cortés JA, Lucena León C, Zarco-Tejada PJ. High-resolution hyperspectral and thermal imagery acquired from UAV platforms for early detection of *Verticillium* wilt using fluorescence, temperature and narrow-band índices. 7. 2014. 10.5880/TR32DB.KGA94.3.

[4] Calderón R, Navas-Cortés JA, Lucena C, Zarco-Tejada PJ. High-resolution airborne hyperspectral and thermal imagery for early detection of *Verticillium* wilt of olive using fluorescence, temperature and narrow-band spectral indices. Remote Sens Environ. 2013;139:231–45. 10.1016/J.RSE.2013.07.031.

[5] Camino C, Araño K, Berni JA, Dierkes H, Trapero-Casas JL, León-Ropero G, Montes-Borrego M, Roman-Écija M, Velasco-Amo MP, Landa BB, Navas-Cortes JA, Beck PSA. Detecting *Xylella fastidiosa* in a machine learning framework using Vcmax and leaf biochemistry quantified with airborne hyperspectral imagery. Remote Sens Environ. 2022;282: 113281. 10.1016/J.RSE.2022.113281.

[6] Campbell PKE, Huemmrich KF, Middleton EM, Ward LA, Julitta T, Daughtry CST, Burkart A, Russ AL, Kustas WP. Diurnal and seasonal variations in chlorophyll fluorescence associated with photosynthesis at leaf and canopy scales. Remote Sensing. 2019;11(5):488. 10.3390/RS11050488.

[7] Chen S, Xu X, Dai X, Yang C, Qiang S. Identification of tenuazonic acid as a novel type of natural photosystem II inhibitor binding in QB-site of *Chlamydomonas reinhardtii*. Biochim Biophys Acta Bioenerg. 2007;1767(4):306–18. 10.1016/J.BBABIO.2007.02.007. 10.1016/j.bbabio.2007.02.007 17379181

[8] Chen S, Yin C, Qiang S, Zhou F, Dai X. Chloroplastic oxidative burst induced by tenuazonic acid, a natural photosynthesis inhibitor, triggers cell necrosis in *Eupatorium adenophorum* Spreng. Biochim Biophys Acta Bioenerg. 2010;1797(3):391–405. 10.1016/J.BBABIO.2009.12.007. 10.1016/j.bbabio.2009.12.007 20026008

[9] Das S, Biswas A, Vimalkumar C, Sinha P. Deep learning analysis of rice blast disease using remote sensing images. IEEE Geosci Remote Sens Lett. 2023;20:1–5. 10.1109/LGRS.2023.3244324.

[10] Dean RA, Talbot NJ, Ebbole DJ, Farman ML, Mitchell TK, Orbach MJ, Thon M, Kulkarni R, Xu JR, Pan H, Read ND, Lee YI, Carbone I, Brown D, Yeon YO, Donofrio N, Jun SJ, Soanes DM, Djonovic S, et al. The genome sequence of the rice blast fungus *Magnaporthe grisea*. Nature. 2005;434(7036):980–6. 10.1038/nature03449. 15846337 10.1038/nature03449

[11] Deb S, Madhavan VN, Gokulan CG, Patel HK, Sonti RV. Arms and ammunitions: effectors at the interface of rice and it’s pathogens and pests. Rice. 2021;14(1):94. 10.1186/S12284-021-00534-4. 34792681 10.1186/s12284-021-00534-4 PMC8602583

[12] Dechant B, Ryu Y, Badgley G, Zeng Y, Berry JA, Zhang Y, Goulas Y, Li Z, Zhang Q, Kang M, Li J, Moya I. Canopy structure explains the relationship between photosynthesis and sun-induced chlorophyll fluorescence in crops. Remote Sens Environ. 2020;241: 111733. 10.1016/j.rse.2020.111733.

[13] Demilie WB. Plant disease detection and classification techniques: a comparative study of the performances. J Big Data. 2024;11(1):1–43. 10.1186/S40537-023-00863-9.

[14] Fisher MC, Henk DA, Briggs CJ, Brownstein JS, Madoff LC, McCraw SL, Gurr SJ. Emerging fungal threats to animal, plant and ecosystem health. Nature. 2012;484(7393):186–94. 10.1038/nature10947. 22498624 10.1038/nature10947 PMC3821985

[15] Frankenberg C, Fisher JB, Worden J, Badgley G, Saatchi SS, Lee JE, Toon GC, Butz A, Jung M, Kuze A, Yokota T. New global observations of the terrestrial carbon cycle from GOSAT: patterns of plant fluorescence with gross primary productivity. Geophys Res Lett. 2011;38(17):L17706. 10.1029/2011GL048738.

[16] Frankenberg C, O’Dell C, Berry J, Guanter L, Joiner J, Köhler P, Pollock R, Taylor TE. Prospects for chlorophyll fluorescence remote sensing from the Orbiting Carbon Observatory-2. Remote Sensing of Environment. 2014;147:1–12. 10.1016/J.RSE.2014.02.007

[17] Gamon JA, Huemmrich KF, Wong CYS, Ensminger I, Garrity S, Hollinger DY, Noormets A, Peñuelask J. A remotely sensed pigment index reveals photosynthetic phenology in evergreen conifers. Proceedings of the National Academy of Sciences of the United States of America. 2016;113(46):13087–92. 10.1073/PNAS.1606162113/SUPPL_FILE/PNAS.201606162SI.PDF 10.1073/pnas.1606162113 PMC5135292 27803333

[18] Gitelson AA, Kaufman YJ, Merzlyak MN. Use of a green channel in remote sensing of global vegetation from EOS-MODIS. Remote Sensing of Environment. 1996;58(3):289–298. 10.1016/S0034-4257(96)00072-7

[19] Gitelson AA, Buschmann C, Lichtenthaler HK. The chlorophyll fluorescence ratio F735/F700 as an accurate measure of the chlorophyll content in plants. Remote Sens Environ. 1999;69(3):296–302. 10.1016/S0034-4257(99)00023-1.

[20] Gitelson AA, Viña A, Ciganda V, Rundquist DC, Arkebauer TJ. Remote estimation of canopy chlorophyll content in crops. Geophys Res Lett. 2005;32(8):1–4. 10.1029/2005GL022688.

[21] Guanter L, Zhang Y, Jung M, Joiner J, Voigt M, Berry JA, Frankenberg C, Huete AR, Zarco-Tejada P, Lee JE, Moran MS, Ponce-Campos G, Beer C, Camps-Valls G, Buchmann N, Gianelle D, Klumpp K, Cescatti A, Baker JM, Griffis TJ. Global and time-resolved monitoring of crop photosynthesis with chlorophyll fluorescence. Proc Natl Acad Sci USA. 2014;111(14):E1327–33. 10.1073/PNAS.1320008111. 24706867 10.1073/pnas.1320008111 PMC3986187

[22] Hornero A, Zarco-Tejada PJ, Quero JL, North PRJ, Ruiz-Gómez FJ, Sánchez-Cuesta R, Hernandez-Clemente R. Modelling hyperspectral- and thermal-based plant traits for the early detection of *Phytophthora*-induced symptoms in oak decline. Remote Sens Environ. 2021;263: 112570. 10.1016/J.RSE.2021.112570.

[23] IRRI. Standard Evaluation System for Rice (4th ed.). 1996.

[24] James WC. Assessment of plant diseases and losses. Annu Rev Phytopathol. 1974;12(1):27–48. 10.1146/ANNUREV.PY.12.090174.000331.

[25] Kalaji HM, Schansker G, Ladle RJ, Goltsev V, Bosa K, Allakhverdiev SI, Brestic M, Bussotti F, Calatayud A, Dąbrowski P, Elsheery NI, Ferroni L, Guidi L, Hogewoning SW, Jajoo A, Misra AN, Nebauer SG, Pancaldi S, Penella C, et al. Frequently asked questions about in vivo chlorophyll fluorescence: practical issues. Photosynth Res. 2014;122(2):121–58. 10.1007/S11120-014-0024-6. 25119687 10.1007/s11120-014-0024-6 PMC4210649

[26] Kimm H, Guan K, Burroughs CH, Peng B, Ainsworth EA, Bernacchi CJ, Moore CE, Kumagai E, Yang X, Berry JA, Wu G. Quantifying high-temperature stress on soybean canopy photosynthesis: the unique role of sun-induced chlorophyll fluorescence. Glob Change Biol. 2021;27(11):2403–15. 10.1111/GCB.15603. 10.1111/gcb.15603 33844873

[27] Kimm H, Guan K, Jiang C, Miao G, Wu G, Suyker AE, Ainsworth EA, Bernacchi CJ, Montes CM, Berry JA, Yang X, Frankenberg C, Chen M, Köhler P. A physiological signal derived from sun-induced chlorophyll fluorescence quantifies crop physiological response to environmental stresses in the U.S. Corn Belt. Environ Res Lett. 2021;16(12): 124051. 10.1088/1748-9326/AC3B16.

[28] Logan BA, Adams WW, Demmig-Adams B. Viewpoint: avoiding common pitfalls of chlorophyll fluorescence analysis under field conditions. Funct Plant Biol. 2007;34(9):853–9. 10.1071/FP07113. 32689413 10.1071/FP07113

[29] Luo X, Croft H, Chen JM, He L, Keenan TF. Improved estimates of global terrestrial photosynthesis using information on leaf chlorophyll content. Glob Change Biol. 2019;25(7):2499–514. 10.1111/GCB.14624. 10.1111/gcb.14624 30897265

[30] Magney TS, Barnes ML, Yang X. On the covariation of chlorophyll fluorescence and photosynthesis across scales. Geophys Res Lett. 2020;47(23): e2020GL091098. 10.1029/2020GL091098.

[31] Magney TS, Frankenberg C, Fisher JB, Sun Y, North GB, Davis TS, Kornfeld A, Siebke K. Connecting active to passive fluorescence with photosynthesis: a method for evaluating remote sensing measurements of Chl fluorescence. New Phytol. 2017;215(4):1594–608. 10.1111/NPH.14662. 28664542 10.1111/nph.14662

[32] Magney TS, Frankenberg C, Köhler P, North G, Davis TS, Dold C, Dutta D, Fisher JB, Grossmann K, Harrington A, Hatfield J, Stutz J, Sun Y, Porcar-Castell A. Disentangling changes in the spectral shape of chlorophyll fluorescence: implications for remote sensing of photosynthesis. J Geophys Res Biogeosci. 2019;124(6):1491–507. 10.1029/2019JG005029.

[33] Mandal N. Characterization of rice blast disease using greenness index, canopy temperature and vegetation indices. Int J Agric Environ Biotechnol. 2022;15(1):81–9. 10.30954/0974-1712.01.2022.10.

[34] Mandal N, Adak S, Das DK, Sahoo RN, Mukherjee J, Kumar A, Chinnusamy V, Das B, Mukhopadhyay A, Rajashekara H, Gakhar S. Spectral characterization and severity assessment of rice blast disease using univariate and multivariate models. Front Plant Sci. 2023;14:1067189. 10.3389/FPLS.2023.1067189. 36909416 10.3389/fpls.2023.1067189 PMC9997726

[35] Miedaner T. Breeding strategies for improving plant resistance to diseases. In: Advances in plant breeding strategies: agronomic, abiotic and biotic stress traits, vol. 2. Cham: Springer; 2016. p. 561–99.

[36] Nelson R, Wiesner-Hanks T, Wisser R, Balint-Kurti P. Navigating complexity to breed disease-resistant crops. Nat Rev Genet. 2017;19(1):21–33. 10.1038/nrg.2017.82. 29109524 10.1038/nrg.2017.82

[37] Oerke E-C. Rice blast disease: Edited by R. S. Zeigler, S. A. Leong and P. S. Teng. CAB International, Wallingford, UK, in association with the International Rice Research Institute, Los Baños, Philippines, 1994. 626 pp. Price: US$135 (hardback). ISBN 0 85198 935 7. Agric Syst. 1996;51(3):367–9. 10.1016/0308-521X(96)86783-7.

[38] Porcar-Castell A, Malenovský Z, Magney T, Van Wittenberghe S, Fernández-Marín B, Maignan F, Zhang Y, Maseyk K, Atherton J, Albert LP, Robson TM, Zhao F, Garcia-Plazaola JI, Ensminger I, Rajewicz PA, Grebe S, Tikkanen M, Kellner JR, Ihalainen JA, et al. Chlorophyll a fluorescence illuminates a path connecting plant molecular biology to Earth-system science. Nat Plants. 2021;7(8):998–1009. 10.1038/s41477-021-00980-4. 34373605 10.1038/s41477-021-00980-4

[39] Porcar-Castell A, Tyystjärvi E, Atherton J, Van Der Tol C, Flexas J, Pfündel EE, Moreno J, Frankenberg C, Berry JA. Linking chlorophyll a fluorescence to photosynthesis for remote sensing applications: mechanisms and challenges. J Exp Bot. 2014;65(15):4065–95. 10.1093/JXB/ERU191. 24868038 10.1093/jxb/eru191

[40] Šebela D, Quiñones C, Cruz CV, Ona I, Olejníčková J, Jagadish KSV. Chlorophyll fluorescence and reflectance-based non-invasive quantification of blast, bacterial blight and drought stresses in rice. Plant Cell Physiol. 2018;59(1):30–43. 10.1093/PCP/PCX144. 29370434 10.1093/pcp/pcx144

[41] Severns PM, Sackett KE, Farber DH, Mundt CC. Consequences of long-distance dispersal for epidemic spread: Patterns, scaling, and mitigation. Plant Disease. 2019;103(2):177–191. 10.1094/PDIS-03-18-0505-FE/ASSET/IMAGES/LARGE/PDIS-03-18-0505-FE_BIO4-1547236935998.JPEG 10.1094/PDIS-03-18-0505-FE 30592698

[42] Shi T, Liu Y, Zheng X, Hu K, Huang H, Liu H, Huang H. Recent advances in plant disease severity assessment using convolutional neural networks. Sci Rep. 2023;13(1):1–13. 10.1038/s41598-023-29230-7. 36759626 10.1038/s41598-023-29230-7 PMC9911734

[43] Silva EA, Gouveia-Neto AS, Oliveira RA, Moura DS, Cunha PC, Costa EB, Câmara TJR, Willadino LG. Water deficit and salt stress diagnosis through LED induced chlorophyll fluorescence analysis in *Jatropha curcas* L. J Fluoresc. 2012;22(2):623–30. 10.1007/S10895-011-0998-9. 22051983 10.1007/s10895-011-0998-9

[44] Sims DA, Gamon JA. Relationships between leaf pigment content and spectral reflectance across a wide range of species, leaf structures and developmental stages. Remote Sens Environ. 2002;81(2–3):337–54. 10.1016/S0034-4257(02)00010-X.

[45] Stuthman DD, Leonard KJ, Miller-Garvin J. Breeding crops for durable resistance to disease. Adv Agron. 2007;95:319–67. 10.1016/S0065-2113(07)95004-X.

[46] Tian L, Wang Z, Xue B, Li D, Zheng H, Yao X, Zhu Y, Cao W, Cheng T. A disease-specific spectral index tracks *Magnaporthe oryzae* infection in paddy rice from ground to space. Remote Sens Environ. 2023;285: 113384. 10.1016/J.RSE.2022.113384.

[47] Tian L, Xue B, Wang Z, Li D, Yao X, Cao Q, Zhu Y, Cao W, Cheng T. Spectroscopic detection of rice leaf blast infection from asymptomatic to mild stages with integrated machine learning and feature selection. Remote Sens Environ. 2021;257: 112350. 10.1016/J.RSE.2021.112350.

[48] Valent B, Chumley FG. Molecular genetic analysis of the rice blast fungus, *Magnaporthe grisea*. Annu Rev Phytopathol. 1991;29(1):443–67. 10.1146/ANNUREV.PY.29.090191.002303. 18479196 10.1146/annurev.py.29.090191.002303

[49] Van Der Tol C, Berry JA, Campbell PKE, Rascher U. Models of fluorescence and photosynthesis for interpreting measurements of solar-induced chlorophyll fluorescence. J Geophys Res Biogeosci. 2014;119(12):2312–27. 10.1002/2014JG002713. 27398266 10.1002/2014JG002713 PMC4852699

[50] Van Wittenberghe S, Amin E, Pascual-Venteo AB, Pérez-Suay A, Tenjo C, Sabater N, van der Tol C, Drusch M, Moreno J. Retrieval of leaf-level fluorescence quantum efficiency and NPQ-related xanthophyll absorption through spectral unmixing strategies for future VIS-NIR imaging spectroscopy. Remote Sensing of Environment. 2014;300;113879. 10.1016/J.RSE.2023.113879

[51] Wang K, Zhu J, Xu X, Li T, Wang X, Warner TA, Cheng T, Zhu Y, Cao W, Yao X, Zhang Z. Quantitative monitoring of salt stress in rice with solar-induced chlorophyll fluorescence. Eur J Agron. 2023;150: 126954. 10.1016/J.EJA.2023.126954.

[52] Wang N, Yang P, Clevers JGPW, Wieneke S, Kooistra L. Decoupling physiological and non-physiological responses of sugar beet to water stress from sun-induced chlorophyll fluorescence. Remote Sens Environ. 2023;286: 113445. 10.1016/J.RSE.2022.113445.

[53] Wong CYS, McHugh DP, Bambach N, McElrone AJ, Alsina MM, Kustas WP, Magney TS. Hyperspectral and photodiode retrievals of nighttime LED-induced chlorophyll fluorescence (LEDIF) for tracking photosynthetic phenology in a vineyard. J Geophys Res Biogeosci. 2024;129(1): e2023JG007742. 10.1029/2023JG007742.

[54] Yeo J, Yeon I, You J, Kim DS, Kimm H. Quantum yield for sun-induced chlorophyll fluorescence (ΦF) captures rice plant dynamics under interplant competition. Remote Sensing of Environment. 2025;320:114655. 10.1016/J.RSE.2025.114655

[55] Yin Y, Zhu J, Xu X, Jia M, Warner TA, Wang X, Li T, Cheng T, Zhu Y, Cao W, Yao X. Tracing the nitrogen nutrient status of crop based on solar-induced chlorophyll fluorescence. Eur J Agron. 2023;149: 126924. 10.1016/J.EJA.2023.126924.

[56] Zarco-Tejada PJ, Berni JAJ, Suárez L, Sepulcre-Cantó G, Morales F, Miller JR. Imaging chlorophyll fluorescence with an airborne narrow-band multispectral camera for vegetation stress detection. Remote Sens Environ. 2009;113(6):1262–75. 10.1016/J.RSE.2009.02.016.

[57] Zeng Y, Badgley G, Dechant B, Ryu Y, Chen M, Berry JA. A practical approach for estimating the escape ratio of near-infrared solar-induced chlorophyll fluorescence. Remote Sens Environ. 2019;232: 111209. 10.1016/J.RSE.2019.05.028.

[58] Zeng Y, Chen M, Hao D, Damm A, Badgley G, Rascher U, Johnson JE, Dechant B, Siegmann B, Ryu Y, Qiu H, Krieger V, Panigada C, Celesti M, Miglietta F, Yang X, Berry JA. Combining near-infrared radiance of vegetation and fluorescence spectroscopy to detect effects of abiotic changes and stresses. Remote Sens Environ. 2022;270: 112856. 10.1016/J.RSE.2021.112856.

[59] Zhang Z, Zhang Y. Solar angle matters: diurnal pattern of solar-induced chlorophyll fluorescence from OCO-3 and TROPOMI. Remote Sens Environ. 2023;285: 113380. 10.1016/J.RSE.2022.113380.

[60] Zhao D, Cao Y, Li J, Cao Q, Li J, Guo F, Feng S, Xu T. Early detection of rice leaf blast disease using unmanned aerial vehicle remote sensing: a novel approach integrating a new spectral vegetation index and machine learning. Agronomy. 2024;14(3):602. 10.3390/AGRONOMY14030602/S1.

[61] Zhao D, Feng S, Cao Y, Yu F, Guan Q, Li J, Zhang G, Xu T. Study on the classification method of rice leaf blast levels based on fusion features and adaptive-weight immune particle swarm optimization extreme learning machine algorithm. Front Plant Sci. 2022;13: 879668. 10.3389/FPLS.2022.879668. 35599890 10.3389/fpls.2022.879668 PMC9120945

[62] Zhuang J, Wang Y, Chi Y, Zhou L, Chen J, Zhou W, Song J, Zhao N, Ding J. Drought stress strengthens the link between chlorophyll fluorescence parameters and photosynthetic traits. PeerJ. 2020;8: e10046. 10.7717/PEERJ.10046. 33024649 10.7717/peerj.10046 PMC7520092

