# Supplementary material for: Earlier quantification of rice blast impact via instantaneous chlorophyll fluorescence
Source: Plant Methods. 2025 Jun 5;21:80. doi: 10.1186/s13007-025-01391-8 (PMC12142957; doi:10.1186/s13007-025-01391-8)
Supplement: Supplementary file 1 — Additional file 1. [file 13007_2025_1391_MOESM1_ESM.docx]

**Supplementary Materials**

Table S1. Rice leaf blast scoring criterion (IRRI System, 1996).
Table S2. A summary of rice blast inoculation progress of Hopyeong (rice blast-susceptible cultivar) and Dasan (rice blast-resistant cultivar) with different inoculation levels (5∙10^3^ conidia/mL and 5∙10^5^ conidia/mL, respectively).
**
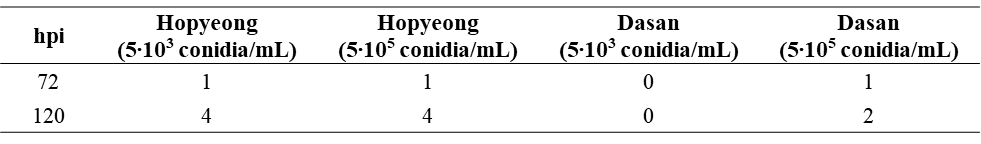
**


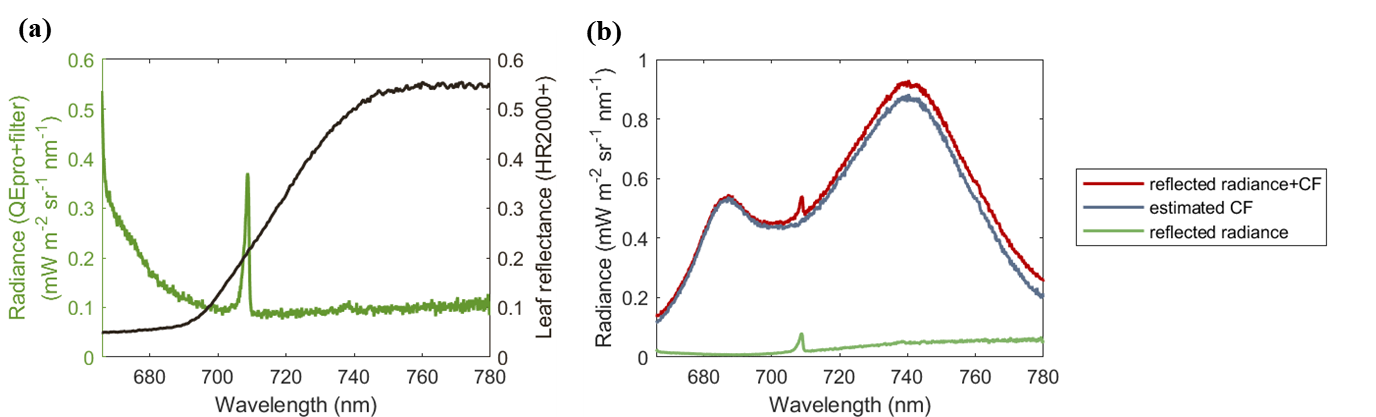


Figure S1. (a) An example of Filter-transmitted radiation from leaf clip measured by QEpro and leaf reflectance obtained by HR2000+. (b) An example of chlorophyll fluorescence containing reflected radiance, reflected radiance, and estimated chlorophyll fluorescence. We removed the portion of estimated reflected radiance (i.e., the product of leaf reflectance and filter-transmitted radiance) from the up-welling radiance from the leaf (i.e., the sum of reflected radiance and CF) measured by QEpro.


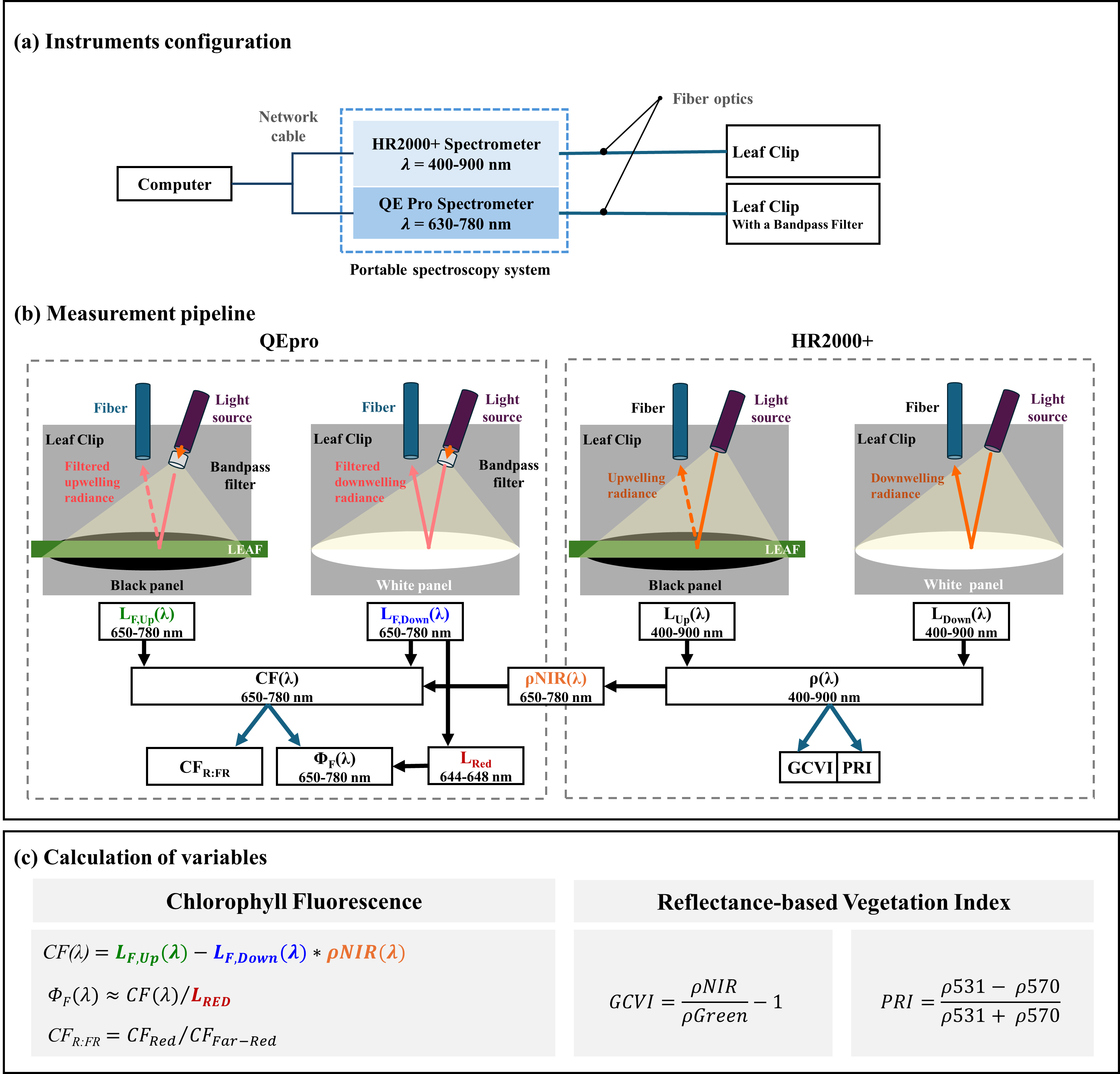


Figure S2. A conceptual diagram for the instruments configuration (a), our hyperspectral measurement pipeline (b), and the calculations of relevant variables (c). *L_Up_* and *L_Down_* are upwelling and downwelling radiance, respectively, and *L_F.Up_* and *L_F.Down_* represent the filtered upwelling radiance and filtered downwelling radiance, respectively. The color of the variables in (b) is corresponding to that in (c).
